# Heritable Thoracic Aortic Diseases in Pediatric Practice: From Molecular Mechanisms to Genotype-Informed Management, a Comprehensive Narrative Review

**DOI:** 10.3390/jcm15145342

**Published:** 2026-07-08

**Authors:** Alessandro Felici, Cristina Angellotto, Arianna Ruta, Mauro Ciro Antonio Rongioletti, Paolo Versacci, Gioia Mastromoro

**Affiliations:** 1 Neonatal and Pediatric Department, Ospedale dei Castelli, 00040 Ariccia, Italy; 2 Department of Maternal Infantile and Urological Sciences, Sapienza University of Rome, 00161 Rome, Italy; cristina.angellotto@uniroma1.it (C.A.); paolo.versacci@uniroma1.it (P.V.); 3 Department of Laboratory Science, Ospedale Isola Tiberina-Gemelli Isola, 00186 Rome, Italygioia.mastromoro@fbf-isola.it (G.M.)

**Keywords:** heritable thoracic aortic disease, pediatric aortopathy, genetic testing, genotype–phenotype correlation, risk stratification, precision medicine

## Abstract

**Background**: Heritable thoracic aortic disease (HTAD) encompass a heterogeneous spectrum of conditions characterized by increased susceptibility to developing thoracic aortic aneurysm and life-threatening complications, including aortic dissection and rupture. Despite distinct underlying mechanisms involving extracellular matrix integrity, vascular smooth muscle cell function, and dysregulation of signaling pathways, these disorders converge on a shared vulnerability of the aortic wall. Although acute events typically occur in adulthood, the disease process often begins early in life, making HTAD highly relevant in pediatric practice, where early recognition and longitudinal management are essential. **Aims**: This narrative review provides a biology- and genetics-oriented, translational complement to current consensus recommendations, framing pediatric HTAD as a developmentally shaped disorder of the aortic wall in which genotype increasingly informs diagnosis, surveillance, and treatment. **Methods**: Relevant studies were identified through a comprehensive PubMed search, with particular focus on pathogenic mechanisms, current clinical guidelines, follow-up strategies and emerging genetic perspectives. **Results**: Genetic testing is emerging as a key tool for the management of HTAD, although its clinical utility remains limited by provisional genotype–phenotype correlations and inconclusive results. Current risk stratification is still mainly based on aortic diameter surveillance, while pharmacological strategies are predominantly extrapolated from Marfan syndrome trials, highlighting important gaps in evidence. **Conclusions**: Genetic advances are expanding management opportunities in HTAD, but their clinical translation remains challenging. Disease-specific risk models integrating genetic and clinical data may improve individualized risk stratification, treatment strategies and clinical outcomes.

## 1. Introduction

Heritable thoracic aortic disease (HTAD) comprise a heterogeneous group of conditions characterized by a genetic predisposition to thoracic aortic aneurysm and life-threatening complications, including aortic dissection and rupture. These disorders can follow Mendelian inheritance, most commonly autosomal dominant but also autosomal recessive or X-linked. Within this spectrum, both syndromic forms (defined by recognizable and often multisystem clinical manifestations) and non-syndromic forms (where aortopathy represents the predominant or sole clinical feature) can be identified, either in familial contexts or through detection of the molecular basis [1].

Despite the biological heterogeneity underlying the different conditions within the HTAD spectrum, a shared hallmark is the intrinsic structural vulnerability of the thoracic aortic wall. This predisposition confers an increased lifetime risk of catastrophic events, which typically manifest in adulthood but are preceded by a prolonged clinically silent phase during which aortic wall damage progressively accumulates. Emerging evidence indicates that this pathological process begins early in life, with structural and functional alterations already detectable during childhood [2,3].

For decades, the management of pediatric aortopathies has relied primarily on guidelines developed for adult patients, with several limitations arising from fundamental differences between age groups. Children exhibit smaller absolute aortic diameters, distinct growth trajectories, and evolving skeletal and cardiovascular manifestations. As a result, this mismatch between available evidence and pediatric clinical practice has led to heterogeneous management strategies across centers. Recently, the publication of the first American Heart Association scientific consensus statement specifically addressing cardiovascular management in children with aortopathies has provided a standardized framework for diagnosis, medical therapy, exercise recommendations, and surgical indications in both heritable and acquired forms [3]. This approach introduces a gene-centered strategy for risk stratification, representing a shift toward precision medicine [1].

Despite these advances, clinical management continues to rely primarily on serial imaging of aortic dimensions, using dilation as a surrogate marker of aortic wall damage. However, diameter measurement alone may not fully capture disease severity across the heterogeneous spectrum of HTAD, particularly in early stages of the disease, when structural alterations may remain clinically under-recognized. Furthermore, for many rare subtypes, preventive strategies remain largely extrapolated from clinical experience in Marfan syndrome (MFS) rather than grounded in disease-specific evidence [2,3,4].

Although molecularly defined forms of HTAD range from rare to ultra-rare [1,2], their true prevalence is likely underestimated: the substantial burden of acute aortic events occurring at a young age suggests that these conditions may be considerably more common than currently recognized [1]. What unifies them is not their frequency but the severity and preventability of their complications, as aortic dissection and rupture are frequently lethal, disproportionately affect young patients, and can often be averted through timely recognition and surveillance, a consideration particularly relevant to pediatric care.

Building on this rapidly evolving field, this review is conceived as a translational, biology- and genetics-oriented complement to the 2024 AHA scientific statement on cardiovascular management of aortopathy in children [3] and to existing reviews. Rather than reiterating diagnostic and management recommendations, which are addressed concisely and cross-referenced to the consensus, it examines the biological and genetic rationale needed to interpret the heterogeneous HTAD spectrum as it emerges and evolves during childhood. Its added value lies in framing pediatric HTAD as a developmentally shaped disorder of the aortic wall, in which early structural damage and genotype-specific wall vulnerability may precede the dimensional changes on which surveillance currently relies, making childhood and adolescence a critical yet still incompletely characterized window for risk stratification. Beyond current testing indications, the review also critically examines the limitations of genetic testing techniques and molecular result interpretation, and how genetic information is beginning to inform surveillance and therapeutic decisions despite the still-limited genotype-specific pediatric evidence.

### Materials and Methods

Literature search and study selection were conducted independently by two investigators, and discrepancies in study selection were resolved through discussion until consensus was reached. Relevant articles for the narrative literature review were identified through a PubMed search performed up to May 2026, without restriction on publication date. Search terms included combinations of the following keywords: “heritable thoracic aortic disease”, “pediatric aortopathy”, “children”, “genetics”, “non-syndromic thoracic aortopathy”, “genetic testing”, “genotype–phenotype correlation”, “Marfan syndrome”, “Loeys-Dietz syndrome”, “aortic aneurysm”, “aortic dissection”, “Z-score”, “aortic stiffness”, “biomarkers”, “medical therapy” and “surgery”. Additional studies were identified through manual screening of reference lists from relevant publications, recent consensus statements, and review articles. Studies were included if they addressed the genetic basis, pathophysiology, diagnosis, risk stratification, surveillance, or management of HTAD. Evidence was prioritized in the following order: pediatric clinical data; adult clinical data; and preclinical studies where clinical evidence was lacking. Non-English publications, conference abstracts without an available full text, and isolated case reports without generalizable mechanistic or clinical insight were excluded. When discussing clinical implications, evidence was weighted according to study design, prioritizing randomized controlled trials and meta-analyses, followed by prospective studies with robust methodology, with observational retrospective data considered when they represented the only available evidence. This corresponds to a structured but non-systematic search strategy, consistent with the narrative design of the review.

## 2. Molecular Mechanisms Underlying Heritable Thoracic Aortic Diseases

The thoracic aorta accommodates the entire cardiac output while buffering the pulsatile load generated by ventricular ejection. Its wall is continuously exposed to elevated pressure and cyclic mechanical forces, including radial stretch and shear stress, requiring both structural resilience and dynamic adaptability. Rather than acting as a passive conduit, the aorta actively stores kinetic energy during systole and releases it during diastole, maintaining continuous downstream perfusion through the ‘Windkessel effect’ [2]. The histological organization of the aortic wall is specialized to sustain repetitive mechanical stress [5]. At the core of this system lies a tightly integrated elastic-contractile unit, in which a load-bearing extracellular matrix (ECM) is functionally coupled with vascular smooth muscle cells (VSMCs). The ECM provides tensile strength and elastic recoil, while VSMCs actively regulate wall tone and matrix turnover. Through mechanosensing processes coupling the extracellular environment to the cytoskeleton, VSMCs continuously adapt their contractile state and synthetic activity in response to mechanical stimuli [6,7]. Within the tunica media, this organization is structured into lamellar units, composed of elastic lamellae interspersed with layers of VSMCs and arranged concentrically along the vessel circumference. The number of lamellar units increases with vessel diameter and local hemodynamic load, reflecting developmental adaptation to mechanical stress, with the highest density observed in the ascending thoracic aorta [8].

HTAD encompasses a spectrum of conditions resulting from genetically determined disruption of this delicately balanced system, culminating in structural fragility of the aortic wall. Despite marked phenotypic heterogeneity, these conditions can be conceptually grouped, based on pathogenic mechanism, into three main categories: (1) ECM defects and loss of medial structural integrity; (2) VSMC dysfunction and impaired contractility; and (3) defective cell–matrix interactions and mechanotransduction [2,9,10].

### 2.1. Extracellular Matrix Structural Defects

Defects of the ECM structural components or their assembly and stabilization compromise the mechanical integrity of the aortic wall, predisposing to progressive mechanical weakening. Monoallelic pathogenic variants in *FBN1*, encoding fibrillin-1, disrupt microfibril formation required for elastin deposition. This results in defective elastic fiber assembly and reduced mechanical integrity of the aortic media, a hallmark of MFS [11]. Histologically, this manifests as fragmentation of elastic lamellae, loss of lamellar organization, and accumulation of proteoglycans within the media [2].

Biglycan, encoded by *BGN*, is a small leucine-rich proteoglycan that contributes to ECM organization through interactions with collagen and elastin. Loss-of-function variants have been associated with Meester–Loeys syndrome, a syndromic form of HTAD [12,13]. Importantly, both fibrillin-1 and biglycan also regulate Transforming Growth Factor-β (TGF-β) bioavailability, linking ECM structural defects to downstream signaling dysregulation (discussed in Section 2.3).

Monoallelic pathogenic variants in *COL3A1*, clinically associated with vascular Ehlers–Danlos syndrome vascular type (vEDS), impair the synthesis and stable assembly of type III collagen, a major structural component of the arterial wall [14].

Defects in proteins involved in elastic fiber formation and maturation also cause severe aortopathies. Biallelic variants in *EFEMP2*, encoding fibulin-4, impair elastic fiber formation and crosslinking within the media, leading to severe arterial fragility with diffuse aneurysmal dilation, tortuosity, and stenosis from early infancy [15]. Similarly, biallelic variants in *SLC2A10*, encoding the facilitative glucose transporter GLUT10, disrupt proteoglycan synthesis and elastic fiber organization in the arterial wall [16].

Monoallelic pathogenic variants in *LOX*, encoding lysyl oxidase, impair the oxidative crosslinking of collagen and elastin fibers, reducing matrix mechanical resilience [17]. Defects in ECM structure and assembly represent a central mechanism underlying several forms of HTAD, primarily affecting elastic fiber integrity and medial architecture.

### 2.2. Vascular Smooth Muscle Cell Dysfunction

VSMCs are central to the maintenance of medial integrity through active force generation, vascular tone adaptation, and ECM turnover regulation. Several HTAD-associated genes encode components of the smooth muscle contractile apparatus or its regulatory pathways, and their disruption leads to impaired force transmission and mechanotransduction.

Monoallelic pathogenic variants in *ACTA2*, encoding smooth muscle α-actin, the most abundant protein in differentiated VSMCs, impair actin filament polymerization and disrupt force transmission within the contractile unit [18]. *ACTA2* represents the most frequent genetic cause of non-syndromic HTAD, accounting for 10–14% of familial cases. Histologically, *ACTA2*-related aortopathy is characterized by VSMC disorganization, medial fibrosis, and reduced contractile filament density [19]. Notably, *ACTA2* displays remarkable intragenic phenotypic heterogeneity. While most pathogenic variants cause non-syndromic aortopathy, specific variants in codon 179 cause multisystemic smooth muscle dysfunction syndrome (SMDS) [20,21]. Variants in *MYH11*, encoding smooth muscle myosin heavy chain, impair the interaction between myosin thick filaments and actin, resulting in defective force generation. *MYH11*-related aortopathy is associated with thoracic aortic aneurysm, patent ductus arteriosus, and, in some cases, cerebrovascular involvement [22]. Loss-of-function variants in *MYLK* reduce phosphorylation of the regulatory light chain of myosin, impairing VSMC contractile activation and reducing force generation, thereby decreasing the ability of the aortic wall to withstand physiological hemodynamic stress [23,24]. In contrast, gain-of-function variants in *PRKG1*, of which c.530G>A; p.Arg177Gln is the most common, lead to constitutive, cGMP-independent activation of protein kinase G, resulting in persistent VSMC relaxation through reduced myosin light chain phosphorylation. This aberrant signaling impairs the ability of the aortic wall to respond to mechanical stimuli, compromising stress resilience [25]. Histologically, these conditions share some common features, including elastic fiber fragmentation and VSMC loss. However, *PRKG1*-related aortopathy may exhibit a more dynamic and stress-dependent remodeling profile, consistent with constitutive activation of cGMP signaling pathways [25,26].

Finally, mechanosensing defects can also arise from abnormalities in cytoskeletal anchoring proteins that mediate the physical linkage between the intracellular contractile apparatus and the ECM. Emerging evidence suggests that loss-of-function variants in *FLNA*, encoding filamin-A, may impair the linkage between the actin cytoskeleton and transmembrane integrins, thereby altering cellular mechanosensing and promoting abnormal vascular remodeling [27,28].

### 2.3. Dysregulation of the TGF-β Signaling Pathway: A Converging Mechanism

The TGF-β signaling pathway acts as a central hub in HTAD pathogenesis: both primary pathway defects and ECM abnormalities affecting TGF-β bioavailability converge on dysregulated signaling, which in turn drives medial degeneration through ECM remodeling and VSMC dysfunction. TGF-β signals through both canonical (SMAD2/3-dependent) and non-canonical (ERK1/2, p38 MAPK) pathways, with the latter implicated as important drivers of aneurysm development in experimental models [29,30].

In MFS, deficient fibrillin-1 fails to sequester latent TGF-β complexes within the ECM, resulting in increased bioavailability of active TGF-β and excessive downstream signaling, which promotes matrix metalloproteinase (MMP) activation, elastin degradation, and pathological collagen remodeling [2,31]. Similarly to fibrillin-1, biglycan also modulates the TGF-β signaling pathway; *BGN* loss-of-function variants are associated with increased TGF-β signaling activity, further linking ECM defects to dysregulated signaling pathways [12,13]. In Loeys–Dietz syndrome (LDS), heterozygous loss-of-function variants in genes encoding TGF-β receptors (*TGFBR1*, *TGFBR2*), downstream effectors (*SMAD2*, *SMAD3*), or ligands (*TGFB2*, *TGFB3*) paradoxically result in enhanced TGF-β signaling within aneurysmal tissue, as demonstrated by increased nuclear phospho-SMAD2 accumulation [32,33]. Similarly, gain-of-function variants in *SKI* cause Shprintzen–Goldberg syndrome through loss of transcriptional repression of TGF-β activity [34].

**Figure 1:**
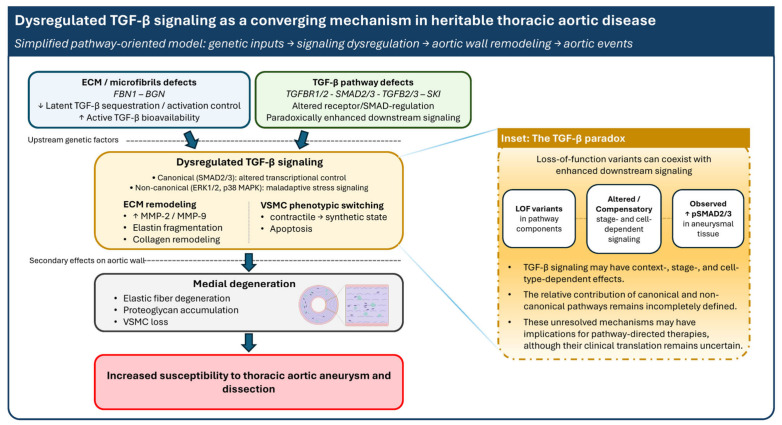
Dysregulated TGF-β signaling as a converging mechanism in HTAD. Distinct genetic defects affecting ECM integrity or TGF-β pathway components converge on altered canonical and non-canonical signaling, driving ECM remodeling, VSMC dysfunction, medial degeneration, and susceptibility to aortic aneurysm and dissection. The inset depicts the TGF-β paradox, in which loss-of-function variants coexist with enhanced downstream signaling. Abbreviations: *BGN*, biglycan; ECM, extracellular matrix; ERK, extracellular signal-regulated kinase; *FBN1*, fibrillin-1; HTAD, heritable thoracic aortic disease; LOF, loss of function; MAPK, mitogen-activated protein kinase; MMP, matrix metalloproteinase; pSMAD, phosphorylated SMAD; *SKI*, SKI proto-oncogene; SMAD, suppressor of mothers against decapentaplegic homolog; TGF-β, transforming growth factor-β; TGFB2/3, transforming growth factor-β 2/3; TGFBR1/2, transforming growth factor-β receptor 1/2; VSMC, vascular smooth muscle cell.

Beyond maladaptive ECM remodeling, dysregulated TGF-β signaling has been linked to VSMC phenotypic modulation, altered contractile-marker expression, apoptosis, and senescence. These observations should not be interpreted as evidence that TGF-β signaling is uniformly deleterious. Rather, experimental studies indicate that basal TGF-β signaling in vascular smooth muscle cells is required for postnatal aortic wall homeostasis, whereas excessive, compensatory, or temporally inappropriate pathway activation may contribute to aneurysm progression in selected disease contexts [35,36]. Thus, the apparent “TGF-β paradox” in HTAD is best viewed as a context-dependent uncoupling between impaired proximal pathway components and increased downstream signaling markers in diseased aortic tissue [37]. The relative contributions of canonical SMAD-dependent signaling and non-canonical MAPK pathways, together with their cell-type-, genotype-, and stage-specific effects, remain incompletely resolved and have direct implications for pathway-directed therapies [30,36,38]. A simplified pathway-oriented overview of these converging mechanisms, including the context-dependent and unresolved aspects of the TGF-β paradox in heritable thoracic aortic disease, is provided in Figure 1.

Collectively, these pathogenic mechanisms converge in a loss of medial structural integrity, resulting in impaired load-bearing capacity of the aortic wall and increased susceptibility to progressive dilation and dissection. Despite these shared mechanisms and histopathological features of medial degeneration, including elastic fiber fragmentation, VSMC loss, and proteoglycan accumulation, increasing evidence indicates that the pattern and progression of aortic damage are largely gene-specific [2,38].

## 3. Disease Progression: Early-Life Determinants and Limitations of Current Risk Models

### 3.1. Disease Progression in Pediatric Patients

The natural history of HTAD is difficult to define due to the rarity of these conditions; moreover, their heterogeneity precludes broad generalizations. Historically, aortic dissection has been the leading cause of mortality in MFS, accounting for up to 80% of deaths and typically occurring in the fourth or fifth decade of life; however, the implementation of early diagnosis, surveillance, and preventive strategies has significantly improved outcomes, with life expectancy now approaching 60–70 years in recent cohorts [39,40].

Although acute aortic events typically occur in adulthood, accumulating evidence indicates that the underlying disease process may begin much earlier. Severe, early-onset and rapidly progressive forms of MFS may present with aortic dilation in infancy, early childhood, and occasionally even prenatally [41]. These forms are enriched for specific *FBN1* variants, particularly missense or in-frame variants clustered within the central “neonatal region” of *FBN1*, classically encompassing exons 24–32, with a higher prevalence in exons 25 and 26 [42,43]. This represents one of the most robust, though not absolute, genotype–phenotype correlations among the fibrillinopathies. A similar gene- and variant-specific paradigm applies to Loeys–Dietz syndrome (LDS), in which aortopathy accounts for most disease-related morbidity and mortality, but the vascular course is highly heterogeneous; *TGFBR1*- and *TGFBR2*-related disease shows the highest burden of childhood-onset aortic events [44,45]. In detail, specific recurrent missense variants—particularly *TGFBR2* p.Arg528, together with *TGFBR2* p.Arg537—are associated with an earlier and more aggressive course of aortic disease, supporting comprehensive vascular surveillance beginning in childhood [3,4]. Among non-syndromic HTAD, severe and childhood-onset vascular disease is particularly associated with *ACTA2* p.Arg179 variants [4,21,46]. *MYLK*- and *PRKG1*-related aortopathy carry a substantial lifetime risk of acute aortic events, but childhood onset is uncommon: events typically occur in adulthood for *MYLK*, and rise markedly from late adolescence and young adulthood for *PRKG1* [4,25,47].

Beyond these genotype-specific differences, a further layer of vulnerability is shared across the spectrum: the developing aorta is exposed to physiological growth and maturation. Elastin synthesis occurs predominantly during late fetal life and the first years of postnatal life, leaving structural damage to the elastic architecture largely irreversible once this developmental window has passed [48]. During childhood and adolescence, physiological changes including somatic growth, increasing blood pressure, and rising stroke volume progressively augment mechanical stress on the aortic wall [49]. In genetically susceptible individuals, the combined effects of these factors may accelerate medial degeneration and aortic remodeling. In MFS, pre-puberty and puberty represent periods of accelerated aortic root growth, during which the indexed growth rate diverges from that observed in healthy individuals [41,50]. Although comparable longitudinal data are limited for other HTAD conditions, similar biomechanical forces are expected to operate across genotypes, supporting current recommendations for intensified surveillance during periods of rapid growth [3,51].

Taken together, these observations support the concept that aortic wall damage in HTAD begins in early life and may accelerate during periods of rapid growth. In this context, early diagnosis represents a critical opportunity to initiate surveillance before clinically overt manifestations emerge.

### 3.2. Insights from Animal Models: Early-Life Vulnerability and Disease-Specific Wall Damage Progression

Although longitudinal human cohort data documenting early-life progression of aortic wall damage remain limited for many rare conditions within the HTAD spectrum, experimental animal models provide compelling evidence that childhood may represent a critical window for disease initiation and progression. In *Col3A1* haploinsufficient mice, fragmentation of the internal elastic lamina begins early in life and worsens over time [52]. Similarly, *Prkg1*-mutant mice exhibit a normal aortic phenotype at birth, but by 12 months, develop elastin fiber fragmentation, VSMC loss, and medial collagen accumulation [26]; in this model, ascending aortic degeneration is further exacerbated by increased pressure-induced wall stress. In *Smad3*-deficient mice, progressive aortic dilation has been associated with age-dependent inflammatory cell recruitment, elastic fiber disruption, and medial thickening [53]. Evidence from *Lox* heterozygous mice indicates that pathological changes may not be evident at baseline, with inflammatory infiltration and ascending aortic dilation emerging primarily in response to hypertensive stress [54]. Likewise, in *Myh11*-mutant mice, hypertension can precipitate aortic dissection and rupture even in the absence of prior aortic dilation, despite underlying abnormalities in elastin–contractile unit architecture [55]. Mutant mouse models for *Lox* and *Myh11* illustrate a pathogenetic paradigm in which a genetically determined predisposition evolves into aortic wall damage in response to additional stressors (hypertension).

Collectively, these findings suggest that disease progression across the HTAD spectrum is not uniformly linear but may instead be variable and influenced by specific mechanical or biological stressors. Nonetheless, structural alterations of the aortic wall appear to accumulate over time, likely originating in early life and progressively increasing the risk of major aortic events in later disease stages.

### 3.3. Limitations of the Aortic Size-Based Risk Paradigm

In this context, the paradigm according to which aortic fragility manifests as progressive dilation, with a consequent risk of acute aortic events proportional to aortic diameter, is based on observations derived from MFS, the most prevalent and extensively studied HTAD model [2]. This paradigm does not appear to be generalizable to the entire spectrum of HTAD for several reasons. In LDS, aortic involvement appears more heterogeneous, with variable rates of aortic growth and frequent association with aortic and major arterial tortuosity. Acute events may occur not only at the level of the aortic root, but also throughout the thoracic aorta and its major branch vessels, even in the presence of only modest aortic dilation [56,57].

In disorders primarily affecting VSMC contractility, such as *ACTA2* or *MYLK* variants, dissections may occur at relatively modest aortic diameters [58,59]. Similar observations have been reported in *PRKG1*-related aortopathy and vEDS, in which arterial rupture or dissection may occur without prior marked aneurysmal dilation [25,60,61]. Notably, in a recent longitudinal cohort study of a predominantly adult Dutch vEDS population, earlier major vascular events were associated with more pronounced syndromic features, whereas no clear correlation was observed between dissections and aortic diameter [62]. These observations challenge the assumption that diameter is a universal indicator of aortopathy progression and severity. However, despite these limitations, aortic dimensions remain central to current risk assessment strategies, although their predictive value varies across different HTAD conditions.

## 4. Bicuspid Aortic Valve-Associated Aortopathy

Bicuspid aortic valve (BAV)-associated aortopathy is the most common form of aortopathy in pediatric practice. Although its pathogenesis and inheritance differ substantially from those of monogenic HTAD, a subset of patients—particularly those with early-onset, root-predominant dilation—lies at the interface with the heritable spectrum, justifying its inclusion in a genetically oriented review.

BAV is the most prevalent congenital heart defect, affecting 0.5–2% of the general population with a male predominance (M:F ≈ 3:1) [2,63]. Aortic dilation has been reported in up to 50% of pediatric BAV cohorts [3,64]. Unlike monogenic HTAD, BAV usually follows a complex, non-Mendelian pattern of inheritance, with marked familial aggregation and an approximately seven-fold increased risk among first-degree relatives [65]. Its genetic background is thought to be predominantly oligogenic or polygenic, with incomplete penetrance; variants in genes such as *NOTCH1*, *GATA5*, and *SMAD6* have been implicated, although they explain only a minority of cases [66].

Pathogenetically, BAV-associated aortopathy reflects the interplay between intrinsic aortic wall vulnerability and altered hemodynamic stress [63,67]. Histopathological studies show medial degeneration even in non-dilated aortas, supporting an inherent structural defect, while abnormal valve geometry generates eccentric flow and region-specific wall shear stress that drive heterogeneous remodeling [68,69]. This translates into distinct phenotypes, as defined in the 2021 international consensus [70]: an ascending phenotype (the most common, ~70%), a root phenotype (~20%), and an extended phenotype. The root phenotype tends to present earlier, is more frequently observed in younger patients, and may reflect a stronger contribution of genetic determinants [64,71].

Approximately 4–10% of patients with HTAD have a BAV, and up to one quarter of those with BAV and aortic dilation show familial clustering consistent with a heritable trait [1,65]; an increased prevalence of BAV has also been described in carriers of *FBN1* or TGF-β pathway variants [1,2]. Accordingly, BAV may be regarded as part of the HTAD spectrum in selected cases—especially with early-onset dilation, root involvement, or a family history of aortic events—whereas the majority remain mechanistically and genetically distinct. This distinction is clinically actionable, as it informs which children with BAV warrant genetic evaluation rather than valve-focused follow-up alone [3].

The natural history of BAV-associated aortopathy is highly variable. Aortic dilation may begin in childhood even without significant valvular dysfunction, supporting a contribution from intrinsic wall fragility beyond hemodynamic stress alone [3,64]. Reported aortic growth rates, largely derived from adult cohorts, are generally low but vary according to BAV phenotype and clinical subgroup [70,72]; however, pediatric data stratified by aortopathy phenotype, valve morphology, and genetic background remain limited [64]. Although acute aortic events are rare during childhood, the potential for early and progressive aortic dilation justifies structured longitudinal surveillance and, when appropriate, medical or surgical interventions, particularly in patients with coarctation, rapid aortic growth, significant valve dysfunction, or features suggestive of an underlying heritable aortopathy [3].

## 5. Genetic Testing in Pediatric HTAD

### 5.1. From Genetic Diagnosis to Genotype-Informed Management

In the pediatric setting, genetic testing has become integral to both etiological diagnosis and clinical management of HTAD. In selected cases, early molecular characterization can refine risk stratification by guiding genotype-oriented imaging surveillance, structured cascade screening of at-risk relatives, and anticipatory surgical planning, thereby helping to prevent major aortic events [4,73]. More broadly, this evolution reflects the transition of cardiovascular genetics from a primarily adjunctive diagnostic resource to a core instrument for prognostic assessment and therapeutic decision-making, especially when aortic risk cannot be reliably predicted by vessel diameter alone (discussed in Section 3.2) [74]. Therefore, in pediatric HTAD, genetic results can act as a dynamic component of clinical care, capable of shaping longitudinal follow-up, informing family-based prevention, and enabling timely, individualized decision-making, particularly during childhood when early identification offers the greatest potential impact.

The genetic and clinical features of syndromic and non-syndromic HTAD are summarized in Table 1 and Table 2, respectively.

### 5.2. Genetic Testing Strategies in Pediatric HTAD

Genetic testing should be considered in cases where evidence of a molecular cause may alter management. When aortopathy is identified, it is recommended that the child undergo clinical genetic evaluation. Comprehensive family history assessment and careful phenotypic evaluation for syndromic features are essential to guide appropriate genetic testing in patients with suspected HTAD [75].

The 2024 AHA scientific statement on cardiovascular management of aortopathy in children provides a structured framework for genetic testing indications, distinguishing between patients with and without BAV [3]. Indications shared across both groups include syndromic features suggestive of a connective tissue disorder, a personal history and/or a first-degree family history of aortic or arterial dissection or spontaneous bowel perforation, and a first-degree relative carrying a known pathogenic variant in an HTAD gene. In patients without BAV, genetic testing is additionally indicated at lower echocardiographic thresholds for aortic dilation, reflecting the higher pre-test probability of monogenic HTAD in this subgroup [3]. In all cases, when feasible, genetic testing should be initiated in the family member with the most severe phenotype or earliest disease onset to maximize diagnostic yield and facilitate variant interpretation [3,73,76]. Increasingly, incidental aortic abnormalities detected on imaging performed for unrelated reasons are also recognized as triggers for genetic referral [77,78].

Multigene next-generation sequencing (NGS) panels are the current first-line strategy for most patients with suspected HTAD. According to ClinGen, eleven genes have definitive or strong evidence for HTAD: *FBN1*, *TGFBR1*, *TGFBR2*, *SMAD3*, *TGFB2*, *COL3A1*, *ACTA2*, *MYH11*, *MYLK*, *LOX*, and *PRKG1*. These genes are clinically actionable and constitute the minimal core of diagnostic panels [78,79]. Evidence-based gene selection is essential to minimize overtesting and variants of uncertain significance (VUS) [79,80]. Broader panels may include genes with moderate or limited evidence, potentially complicating variant interpretation. According to the American College of Medical Genetics and Genomics (ACMG) criteria, variants in candidate genes may indeed be reported as VUS and not classified as pathogenic or likely pathogenic. Additional candidate genes with emerging or limited evidence are listed in Table 2.

**Figure 2:**
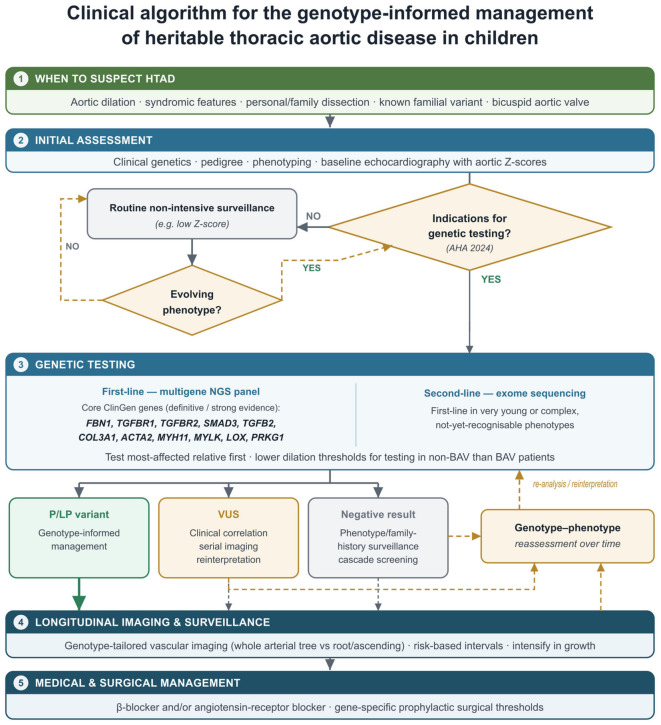
Clinical algorithm for the genotype-informed management of pediatric HTAD. Genetic testing follows the 2024 AHA indications [3] and is interpreted iteratively together with phenotype, family history, and serial imaging, with periodic genetic reassessment. Abbreviations: AHA, American Heart Association; BAV, bicuspid aortic valve; ClinGen, Clinical Genome Resource; HTAD, heritable thoracic aortic disease; NGS, next-generation sequencing; P/LP, pathogenic/likely pathogenic; VUS, variant of uncertain significance.

When strong clinical suspicion persists despite negative first-line targeted gene panel testing, complementary techniques should be integrated into the diagnostic workflow [81,82], and exome sequencing (ES) may be considered as a second-line approach in selected cases [83]. Conversely, in children with complex or evolving phenotypes, especially in very young patients in whom specific syndromic patterns may not yet be clinically recognizable, ES may be appropriate as a first-line strategy, as it can identify pathogenic variants in genes associated with disorders that have not yet fully manifested clinically [75]. Secondary findings detected through ES require careful consideration, particularly in pediatric patients. Pathogenic or likely pathogenic variants in actionable genes defined by the ACMG are identified in approximately 2–3% of cases [84]. Incidental findings, unrelated to the clinical suspicion, are reported in 3–5% of individuals undergoing ES [85]. A further advantage of exome-based approaches is the possibility of reanalyzing raw data over time without the need for repeat sequencing. Figure 2 places these genetic testing strategies within the overall genotype-informed clinical pathway, from initial suspicion through surveillance and management.

The diagnostic yield of genetic testing in HTAD varies considerably, reflecting differences in phenotypic definition, family history, and cohort ascertainment. In an early panel-based series, Campens et al. reported an overall 25.5% detection rate in 264 unrelated probands, with higher yields in clinically enriched MFS and lower yields in isolated non-syndromic disease [86]. In a separate 10-gene panel cohort, Wooderchak-Donahue et al. identified pathogenic or likely pathogenic variants in 10.3% of individuals, whereas VUS were observed in 18.3%, underscoring the interpretive burden of broader testing [80]. These estimates derive largely from cohorts not restricted to children; more recent data from a dedicated pediatric HTAD clinic reported a diagnostic yield of 21%, suggesting that phenotypic enrichment and specialist assessment improve detection rates [87]. Copy number variants (CNVs) also appear clinically relevant, as a meaningful proportion of pathogenic findings may be missed when only conventional sequencing is performed [88].

Genetic testing in pediatric HTAD has implications extending beyond the individual child, directly triggering familial cascade screening. Pre- and post-test genetic counseling plays a critical role in addressing possible and unexpected outcomes, including inconclusive or negative results that do not exclude a heritable condition, as well as the possibility of medically actionable secondary findings when ES is performed.

### 5.3. Challenges in Genetic Interpretation and Remaining Knowledge Gaps

Despite major advances in molecular diagnostics, interpreting HTAD-associated variants remains challenging. Factors such as reduced penetrance, age-dependent expressivity, intrafamilial variability, and the persistent burden of VUS limit the direct translation of molecular findings into unequivocal clinical decisions [74]. Intrafamilial variability and incomplete penetrance are well illustrated in a recent large *TGFBR2* pedigree [89]. This is especially relevant in children, in whom the clinical phenotype may be incomplete at initial evaluation and may evolve over time. In addition, de novo variants are relatively frequent in *FBN1* and in genes of the TGF-β signaling pathway, further complicating counseling and risk assessment [1,79]. Accordingly, genetic findings should be interpreted within an integrated clinical framework rather than as isolated predictors of outcome [73]. Serial imaging, family history, extracardiac manifestations, and longitudinal reassessment remain essential for contextualizing the significance of a molecular finding and tailoring surveillance over time.

VUSs remain among the most problematic findings in current practice. The ACMG/AMP framework provides the general standard for variant classification, but gene-specific refinements are not available for all genes [76,90]. Periodic reinterpretation of genetic results is particularly desirable in children, whose phenotypes may evolve with growth and development [91].

Comparative studies demonstrate that the risk and age at first aortic event differ substantially across genes, reinforcing the rationale for gene-specific surveillance strategies and individualized surgical timing [4,11,45]. However, significant intragenic heterogeneity persists: in *FBN1*, the phenotypic spectrum extends from severe neonatal presentations to relatively isolated aortic dilation, while *ACTA2* may present as isolated non-syndromic aortopathy or as complex multisystem involvement depending on the specific variant [92]. Genotype should therefore inform, but not replace, clinical judgment based on serial imaging, family history, and phenotypic evolution over time.

A substantial proportion of patients with a high clinical suspicion of HTAD remain without an identifiable pathogenic variant [86]. Negative sequencing results in patients with suspected HTAD may reflect several limitations of current molecular testing strategies, including incomplete coverage of coding and regulatory regions, limited sensitivity for CNVs and structural variants, deep intronic or mosaic variants, variants in genes not yet associated with aortic disease, and challenges in the interpretation of rare or non-coding variants [77,79,88]. When results are inconclusive, clinical management should be guided by phenotype and family history [3,93]. Periodic genetic re-evaluation should be considered in light of evolving knowledge and testing strategies, alongside changes in phenotypic expression over time, particularly in pediatric patients in whom growth influences disease manifestation [76,91]. In high-risk families, surveillance imaging of at-risk relatives remains warranted even when no familial pathogenic variant has been identified [3].

### 5.4. Future Directions in Genetic Diagnosis and Risk-Stratification of Pediatric HTAD

Future progress in pediatric HTAD will depend on narrowing the gap between molecular diagnosis and truly individualized risk prediction. Achieving this will require progress along several complementary directions. Clinically relevant differences may arise not only across genes but also among specific variants or molecular mechanisms within the same gene. In this context, variants such as *ACTA2* p.Arg179 and *TGFBR2* p.Arg528 are recognized as high-risk genotypes, with direct implications for surveillance and medical and surgical decision-making [3,77]. For *FBN1*-related Marfan syndrome, however, variant-level stratification has not yet been translated into routine pediatric management [3]. Although genotype–phenotype correlations have been reported, including associations between *FBN1* variant type or location and cardiovascular severity, the available evidence remains largely derived from mixed-age cohorts [94,95].

Large international registries with standardized phenotypic data collection will be essential to support the transition from gene-level to variant-level risk stratification, enabling refinement of surveillance protocols and prophylactic thresholds that are both genotype-informed and developmentally appropriate for children and adolescents.

Population-scale biobank studies suggest that genotype-first screening may identify carriers of pathogenic variants before clinical disease onset, although application to pediatric HTAD remains limited by ethical concerns, age-dependent penetrance, and the lack of prospective data defining long-term risk trajectories [74,96].

Beyond structural variants already detectable through current CNV analysis, non-canonical splice defects and deep intronic alterations represent an under-recognized source of pathogenic variation, and improved detection will likely increase diagnostic resolution in currently unsolved cases [97]. As discussed above, the extension of gene-specific variant classification frameworks and the routine reinterpretation of unresolved findings will further improve the clinical utility of genetic testing over time.

Artificial intelligence is expected to play an increasing role in the management of HTAD by enabling the integration of genomic, imaging, and clinical datasets. Deep-learning approaches applied to cardiovascular imaging have already demonstrated the ability to derive quantitative aortic phenotypes suitable for large-scale genetic analyses in adult populations [98,99]. Moreover, AI-based facial-analysis and automated phenotyping tools may further aid the early recognition of subtle syndromic features [100]. Whether similar strategies can improve risk stratification in pediatric HTAD remains to be established through dedicated prospective studies.

**Figure 3:**
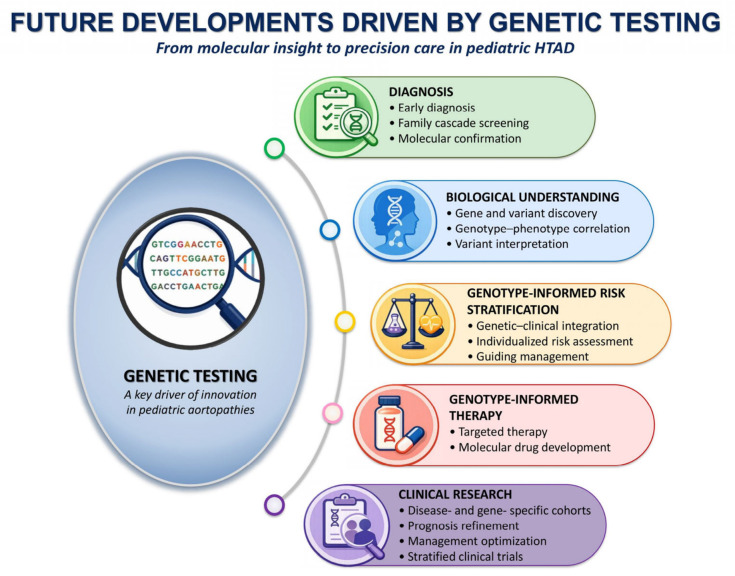
Future developments driven by genetic testing in pediatric aortopathies, spanning diagnosis, biological understanding, risk stratification, therapy, and clinical research.

More broadly, the future of precision care in pediatric HTAD will rest on the integration of genomics with careful phenotyping, advanced imaging, and longitudinal clinical observation. Within this perspective, molecular testing should be viewed not as an isolated diagnostic act, but as one element of an evolving clinical process aimed at anticipating risk, refining surveillance, and improving outcomes across the life course (Figure 3).

## 6. Toward a Multiparametric and Genotype-Driven Management Approach

### 6.1. Z-Score Limitations

Clinical management of pediatric aortopathy is based on serial risk assessment to guide surveillance intervals, medical therapy, lifestyle recommendations, and timing of prophylactic interventions. Alongside genetic characterization, aortic dimensions remain central for clinical decision-making [3]. In children, physiological somatic growth requires normalization of aortic size using Z-scores indexed to body surface area. Despite their widespread use, Z-scores have important limitations. Variability in aortic Z-score assessment partly reflects differences in measurement methodology and reference populations used to derive nomograms. In addition, discrepancies between Z-score models tend to increase at the extremes of the distribution, precisely in the range relevant for defining aortic dilation, highlighting potential limitations in their clinical application [101,102]. This is exemplified in pediatric MFS cohorts, where the proportion of patients classified as having aortic root dilation varies substantially depending on the Z-score equation used [103]. Furthermore, Z-scores are influenced by body size and growth patterns, which may confound interpretation in syndromic conditions characterized by abnormal somatic development [104,105].

To overcome some of these limitations, Z-score models specifically developed for pediatric patients with MFS and related conditions, such as LDS, have been proposed [106]. Although these models remain limited by small sample sizes and genetic heterogeneity, they represent an initial step toward a more tailored approach to aortic dimension assessment and precision-based risk stratification in specific genetic conditions. Future efforts should aim at developing disease-specific or gene-centered nomograms across the spectrum of pediatric HTAD.

A further practical limitation concerns the mismatch between the metrics used for surveillance and those used to guide prophylactic surgery. In children, aortic size is routinely monitored using body-size-adjusted Z-scores, whereas pediatric surgical thresholds are generally expressed as absolute diameters in centimetres, reflecting the absence of validated Z-score-based operative thresholds. This methodological gap has long been recognized and remains unresolved in current pediatric aortopathy recommendations [3,107]. It is particularly relevant in younger or rapidly growing children, in whom a clinically meaningful Z-score elevation may not yet correspond to the absolute diameter conventionally used to trigger intervention.

### 6.2. Cross-Sectional and Functional Aortic Imaging

The aortic involvement in HTAD varies across genotypes not only in severity but also in the pattern of segmental involvement, ranging from isolated aortic root dilation to disease affecting the ascending aorta, aortic arch, or descending aorta [108,109]. In several HTAD subtypes, particularly LDS and vEDS, vascular involvement extends beyond the aorta to include aneurysms of branch vessels, arterial tortuosity, and, in some cases, stenotic or occlusive lesions [33,45]. Similar patterns have also been described in rarer forms such as *EFEMP2*- and *SLC2A10*-related disorders [4]. Assessment of these features often requires cross-sectional imaging of the entire arterial tree, which poses specific challenges in children, including the need for sedation and concerns related to radiation exposure [110]. Genotype-informed imaging strategies are therefore essential to tailor the extent of vascular imaging, while minimizing the diagnostic burden for pediatric patients.

Aortic stiffness is recognized as an important cardiovascular risk factor and tends to increase with age [111]. Non-invasive assessment of aortic stiffness using functional imaging techniques has emerged as a promising approach for the management of thoracic aortic aneurysms, although its role in routine clinical practice remains to be validated [77]. In the context of HTAD risk assessment, functional measures of aortic wall mechanics may provide a useful complement to diameter monitoring. A recent meta-analysis including 1925 mostly adult or mixed-age patients with MFS demonstrated that reduced aortic distensibility is detectable even in vessels with normal diameters, particularly at the aortic root and ascending aorta, suggesting that biomechanical alterations may precede overt morphological dilation [112]. Among the stiffness indices evaluated, the β-stiffness index emerged as a particularly sensitive marker, being elevated across multiple aortic segments in non-dilated aortas and correlating with the rate of subsequent aortic dilation. Consistently, a prospective multicenter cardiovascular magnetic resonance study on a mixed-age MFS cohort demonstrated that proximal aortic longitudinal strain independently predicts aortic root growth and aortic events, supporting the incremental prognostic value of functional parameters over dimensional parameters [113].

Evidence in pediatric populations remains more limited but clinically relevant. In a longitudinal study including children and adolescents with MFS (mean age 11 years), Selamet-Tierney et al. showed that elevated aortic stiffness, measured by elastic modulus and stiffness index, was independently associated with disease progression and adverse outcomes, including aortic surgery, dissection, and mortality [114]. Pediatric echocardiographic studies further report reduced aortic root elasticity and strain in MFS even in the absence of significant dilation [115], while 4D-flow CMR studies in LDS demonstrate altered flow patterns and increased regional stiffness that largely overlap with MFS findings [116].

These functional markers may be particularly relevant in HTAD subtypes where the risk of acute events does not correlate linearly with aortic diameter. In adult patients with *MYH11*-related aortopathy, reduced aortic compliance and distensibility have been detected in the absence of significant dilation [117]. Similarly, an ultrafast ultrasound assessment in adult patients with vEDS demonstrated altered dynamic arterial wall biomechanics that may reflect underlying vascular fragility beyond what static dimensions can capture [118]. Although promising, these findings should be interpreted with caution, as they derive from observational studies conducted in relatively small adult cohorts. Evidence also remains scarce or inconclusive for *ACTA2*-, *MYLK*-, and *PRKG1*-related aortopathies. Prospective studies are needed to establish the role of non-invasive stiffness measures in gene-specific risk stratification.

### 6.3. Circulating Biomarkers

Research on circulating biomarkers in HTAD has focused on pathways involved in ECM remodeling and dysregulated signaling, but no marker has yet reached clinical applicability. Plasma TGF-β has been proposed as a marker of disease activity; however, circulating levels are influenced by platelet activation and have not reliably distinguished affected patients from controls [119]. MMPs, particularly MMP-2 and MMP-9, are key mediators of ECM degradation in experimental aneurysm models [120,121]. In humans, circulating MMP-2 levels have been associated with aortic dilation and increased vascular stiffness predominantly in studies of BAV-associated aortopathy [122], but their clinical utility in monogenic HTAD remains unexplored. Among novel candidates, desmosine, a specific degradation product of mature elastin crosslinks, has been investigated as a marker of aortic wall remodeling in MFS. In a post hoc analysis of the AIMS trial, plasma desmosine levels were shown to rise during childhood and peak in early adolescence, with a more pronounced increase in MFS patients compared to controls, while differences disappeared in adulthood [50]. However, baseline desmosine showed only a non-significant trend toward association with subsequent aortic root growth. Although preliminary, these findings suggest that desmosine may reflect early disease activity and deserves further investigation as a potential biomarker in pediatric populations.

Recently, circulating microRNAs (miRNAs) have emerged as promising biomarkers owing to their remarkable stability in plasma and their regulatory role in vascular smooth muscle cell phenotypic switching, extracellular matrix remodeling, endothelial dysfunction, and TGF-β signaling. Several miRNAs, most notably members of the miR-29 family, together with miR-21, miR-145 and the miR-17–92 cluster, have been implicated in the molecular pathogenesis of thoracic aortic aneurysms [123,124], although current evidence derives predominantly from experimental studies and adult cohorts [125].

Beyond individual biomarkers, multi-omics approaches integrating genomics, transcriptomics, epigenomics, proteomics, metabolomics, and single-cell technologies are beginning to provide a more comprehensive understanding of the molecular mechanisms underlying HTAD [99]. By combining these datasets with imaging parameters and genotype information, future studies may identify molecular signatures capable of improving individualized risk prediction and therapeutic stratification. Despite these promising developments, several challenges continue to hinder the translation of circulating biomarkers into routine clinical practice [126]. Most studies have been conducted in small adult cohorts, while pediatric data remain scarce. Additional limitations include marked genetic and phenotypic heterogeneity, lack of standardized sample collection and analytical protocols, absence of age-specific reference ranges, and limited longitudinal validation. Furthermore, no biomarker has consistently demonstrated incremental predictive value beyond established clinical, genetic, and imaging risk factors [127].

### 6.4. Therapeutic and Prophylactic Management: Current Evidence and Future Directions

Current evidence supporting medical therapy in HTAD derives predominantly from studies in adult patients with MFS. β-blockers and angiotensin receptor blockers (ARBs) have both been associated with a reduction in the rate of aortic root dilation [128,129,130]. A recent meta-analysis of seven randomized trials confirmed that ARBs approximately halve the annual rate of increase in the aortic root Z-score, with effects that appear additive to those of β-blockers, supporting the hypothesis that these agents target complementary pathogenic mechanisms: hemodynamic stress reduction for β-blockers and modulation of maladaptive signaling pathways, including TGF-β, for ARBs [131]. However, none of these trials, individually or combined, has demonstrated a significant impact on hard clinical endpoints such as aortic dissection or cardiovascular mortality.

In pediatric practice, the strongest pharmacological evidence concerns *FBN1*-related MFS, in which β-blocker and/or ARB therapy is integrated into longitudinal aortic surveillance and is generally initiated when aortic dilation reaches guideline-based Z-score thresholds or when high-risk features are present [3]. Nevertheless, even in this relatively well-studied subgroup, pediatric evidence remains limited. The Pediatric Heart Network trial showed comparable efficacy between atenolol and losartan in slowing aortic root growth in children and young adults with MFS, without establishing superiority of either strategy [132]. No pediatric-specific randomized evidence is available for HTAD conditions beyond MFS. Accordingly, β-blocker and ARB recommendations have been extended to the other HTAD entities largely by extrapolation rather than on the basis of condition-specific pediatric evidence, and this generalization should therefore be made with caution. While this extrapolation may be pathophysiologically plausible for conditions involving TGF-β signaling pathway dysregulation, such as LDS, its applicability to disorders driven primarily by ECM structural defects or VSMC contractile dysfunction is less clear, as the underlying disease mechanisms may not be directly targeted by conventional MFS-derived therapies. In vEDS, randomized controlled trial data have shown that celiprolol is associated with a reduction in major arterial events [133], and suggest that the addition of irbesartan may further improve outcomes [134], although these studies did not include pediatric patients and tolerability concerns remain.

A critical limitation of current recommendations is the timing of treatment initiation. In children, medical therapy is generally started only after aortic dilation has been documented, typically defined as a Z-score exceeding 2, although earlier treatment may be considered in selected high-risk conditions, notably in high-risk genotypes such as *ACTA2* p.Arg179 and *PRKG1*, regardless of aortic dimensions [3]. This threshold-based approach does not reflect evidence that treatment is most effective once dilation has occurred; rather, it results from the current inability to reliably identify, among genetically susceptible children, those who will develop progressive aortopathy before dilation becomes manifest. Whether earlier, genotype-informed initiation of medical therapy may benefit selected high-risk patients is a biologically plausible but unproven hypothesis that warrants prospective investigation.

Surgical decision-making in pediatric HTAD is increasingly guided by genotype-specific risk profiles. Prophylactic aortic root replacement remains the definitive strategy to prevent dissection in selected high-risk patients and can be performed in young children in specialized centers [135,136]. Dedicated age- and size-adjusted pediatric thresholds have been defined only for the conditions with the strongest evidence: severe early-onset Marfan syndrome and Loeys–Dietz syndrome due to *TGFBR1*/*TGFBR2* variants with high-risk features [3]. For most other subtypes, surgical timing continues to rely on extrapolated adult thresholds and individualized assessment. Earlier intervention at lower aortic diameters is recommended or considered in higher-risk conditions such as *ACTA2*-related aortopathy, whereas in conditions such as vEDS, decision-making remains highly individualized because dissections may occur at small diameters and specific pediatric thresholds are lacking [3,77]. Notably, in *PRKG1*-related aortopathy, intervention is recommended from the age of 17 years even in the presence of mild dilation [3]. Importantly, pediatric-specific surgical evidence remains limited, and reported series describe substantial postoperative morbidity despite low mortality, complicating the derivation of age-specific operative thresholds [136,137]. Table 3 summarizes the main genotype–phenotype correlations with practical implications for pediatric surveillance, vascular imaging, and risk stratification.

Alongside current medical and surgical strategies, preclinical studies are beginning to explore disease-specific therapeutic strategies that go beyond hemodynamic modulation. In MFS murine models, inhibition of matrix metalloproteinases with doxycycline has been shown to reduce elastic fiber degradation and delay aneurysm progression [138,139]. Pharmacological modulation of oxidative stress pathways, including xanthine oxidase inhibition with allopurinol, has attenuated aortopathy in experimental MFS models [140]. Inhibition of the mTOR pathway with rapamycin has reduced aneurysm growth and improved survival in preclinical models, implicating cellular metabolism and vascular remodeling as potential therapeutic targets [141]. Furthermore, dysregulation of the nitric oxide–soluble guanylate cyclase–PRKG axis has been implicated in aortic disease, and its pharmacological modulation has reversed aortopathy in selected experimental models [142]. Finally, emerging genomics-driven approaches, integrating single-cell transcriptomics and computational methods are beginning to identify novel candidate molecular targets for disease-specific and potentially genotype-tailored interventions [143]. While these strategies remain at an early experimental stage, they illustrate a trajectory toward mechanism-based therapeutics that may complement or eventually replace the current empirical, size-driven treatment paradigm.

## 7. Limitations

Several limitations should be acknowledged. First, as a narrative rather than a systematic review, this work does not rely on a predefined, fully reproducible search and selection protocol or on a formal assessment of study quality and risk of bias; although the search approach is described in the Materials and Methods, the synthesis nonetheless reflects the authors’ selection of the available literature and may be subject to selection bias. Second, the marked heterogeneity of HTAD entities and the rarity of many genotypes hinder cross-study comparison and preclude quantitative conclusions for specific disease subgroups. Third, prospective longitudinal data describing the natural history of aortic disease during childhood remain scarce; much of the available evidence is consequently cross-sectional, retrospective, derived from referral-based cohorts, or extrapolated from adult studies and animal models, whose applicability to the developing human aorta is uncertain. Heterogeneity in imaging protocols, outcome definitions, and follow-up duration further limits direct comparison across studies. Finally, gene–disease relationships, genotype–phenotype correlations, and variant classifications in HTAD continue to evolve, so statements concerning genes with emerging evidence and the interpretation of uncertain variants are necessarily provisional. It should also be emphasized that most genotype-specific recommendations for surveillance and treatment currently derive from observational cohorts, registries, and expert consensus rather than from prospective randomized trials. The overall strength of the evidence underpinning genotype-informed pediatric management is therefore limited, with many recommendations resting on low-to-moderate-certainty data and expert opinion.

## 8. Conclusions

Although HTAD conditions share a common predisposition to life-threatening acute aortic events, they represent biologically distinct conditions driven by different pathogenic mechanisms. From a pediatric perspective, these conditions often progress insidiously during childhood, making early identification and accurate risk stratification a persistent clinical challenge. Genetic characterization is progressively transforming the medical and surgical management of affected patients, enabling genotype-tailored surveillance and intervention. Nevertheless, interpretative difficulties persist in the case of inconclusive results and provisional genotype–phenotype correlations. Current risk stratification strategies are largely modeled on the more prevalent conditions, particularly MFS, and rely predominantly on aortic diameter measurements, a parameter that may not adequately capture the progression of aortic wall damage across the full spectrum of HTAD. Functional imaging techniques and circulating biomarkers may, in the future, complement dimensional assessment and improve the ability to detect subclinical disease progression. The integration of these tools into genotype-informed, multiparametric risk models will be essential to improve the precision of risk stratification in pediatric patients with HTAD. Equally important is the development of disease-specific therapeutic strategies, particularly in children, where current pharmacological recommendations are largely extrapolated from adult trials and robust prospective evidence remains lacking. Progress toward individualized care will require prospective, multicenter pediatric registries designed for disease-specific and genotype-stratified longitudinal data collection. By integrating standardized clinical, imaging, and genetic data over time, such registries could clarify how aortic disease develops and progresses across different genotypes during growth. This evidence would be essential to develop and validate genotype-specific risk models that move beyond diameter-based stratification and support more tailored surveillance, timing of intervention, and therapeutic decisions in children with HTAD.

## Figures and Tables

**Table 1:** Syndromic Heritable Thoracic Aortic Diseases.

Syndrome (MIM Number)	Inheritance	Gene	Prevalence	Aortic Features	Extra-Aortic Key Clinical Features
Marfan syndrome (#154700)	AD	*FBN1*	1:5000–1:10,000	Progressive root dilation (pear-shaped); variable ascending involvement	Ectopia lentis, marfanoid habitus, mitral valve prolapse, scoliosis, pectus deformity, dural ectasia
Loeys–Dietz syndrome (overview)	AD	/	1:50,000	Diffuse arterial tortuosity; root/ascending dilation, with variable distal extension aneurysms throughout the arterial tree, including branch vessels; elongated/gothic aortic arch	Hypertelorism, bifid uvula/cleft palate, velvety skin, cervical spine instability, arterial tortuosity. SMAD3 may be associated with early-onset osteoarthritis.
Loeys–Dietz syndrome 1 (#609192)	AD	*TGFBR1*	~20–25% of LDS		
Loeys–Dietz syndrome 2 (#610168)	AD	*TGFBR2*	~55–60% of LDS		
Loeys–Dietz syndrome 3 (#613795)	AD	*SMAD3*	~5–10% of LDS		
Loeys–Dietz syndrome 4 (#614816)	AD	*TGFB2*	~5–10% of LDS		
Loeys–Dietz syndrome 5 (#615582)	AD	*TGFB3*	~1–5% of LDS		
Loeys–Dietz syndrome 6 (#619656)	AD	*SMAD2*	~1–5% of LDS		
Shprintzen–Goldberg syndrome (#182212)	AD	*SKI*	<1:1,000,000	Root/ascending dilation with possible distal extension	Craniosynostosis, developmental delay, skeletal abnormalities, atrial septal defect
Vascular Ehlers–Danlos syndrome (#130050)	AD	*COL3A1*	1:50,000–1:200,000	Diffuse arteriopathy involving aorta, aortic-branch vessels, medium-size and intracranial arteries	Dysmorphisms, intestinal and uterine rupture, thin translucent skin, easy bruising, hypermobility of small joints
Arterial tortuosity syndrome (#208050)	AR	*SLC2A10*	<1:1,000,000	Diffuse arteriopathy; marked tortuosity; aneurysms and stenoses involving the aorta and major branches; pulmonary artery stenosis	Elongated face, skin hyperextensibility, hernias, diaphragmatic defects, keratoconus/myopia
Cutis Laxa type 1B (#614437)	AR	*EFEMP2*	<1:1,000,000	Diffuse aortic involvement; arterial tortuosity; aneurysms and stenoses	Cutis laxa, hernias, pulmonary bullae, pectus excavatum, advanced bone age
Meester–Loeys syndrome (#300989)	X-linked	*BGN*	<1:1,000,000	Root/ascending dilation with possible extra-aortic involvement	Hypertelorism, pectus deformity, joint hypermobility/contractures, mild skeletal dysplasia, ventriculomegaly, malar hypoplasia with gingival hyperplasia
Smooth Muscle Dysfunction Syndrome (#613834)	AD	*ACTA2*	<1:1,000,000	Root/ascending predominant dilation; PDA; diffuse aneurysmal and occlusive arteriopathy; moyamoya-like cerebrovascular disease	Congenital mydriasis, pulmonary hypertension

This table summarizes the main syndromic HTAD entities, emphasizing genetic background, prevalence, aortic involvement, and extra-aortic features relevant to pediatric recognition. Abbreviations: AD, autosomal dominant; AR, autosomal recessive; HTAD, heritable thoracic aortic disease; PDA, patent ductus arteriosus.

**Table 2:** Non-Syndromic Heritable Thoracic Aortic Disease: Genes, Pathogenic Mechanisms and Aortic Phenotypes.

Gene	Inheritance	Variant Effect	Pathogenic Mechanism	Aortic Features	Gene-Disease Validity
*ACTA2* (other than p.Arg179His/Cys/Leu variants)	AD	Loss of function (mostly dominant-negative)	VSMC—Impaired contractility	Root/Ascending thoracic aortic aneurysm/dissection; PDA; occlusive arterial disease (coronary, moyamoya-like)	**Definitive**
*MYH11*	AD	Loss of function (mostly dominant-negative)	VSMC—Impaired contractility	Root/Ascending aneurysm/dissection; PDA	Established
*MYLK*	AD	Loss of function	VSMC—Impaired contractility	Root/ascending aneurysm, possible distal extension	Established
*PRKG1*	AD	Gain of function (p.Arg177Gln)	VSMC—Increased cGMP activity, abnormal relaxation	Mild root/ascending dilation	Established
*LOX*	AD	Loss of function	ECM—Impaired elastin and collagen cross-linking	Root/ascending aneurysm/dissection with possible arch extension	Established
*FLNA*	X-linked	Loss of function	VSMC—Cytoskeletal organization/mechanosensing defect	Root/ascending aneurysm; systemic vascular involvement; PDA; cardiac valvular anomalies	Emerging evidence
*THSD4*	AD	Loss of function	ECM-Impaired microfibril assembly	Root/ascending aneurysm	Emerging evidence
*MAT2A*	AD	Likely loss of function	Metabolic/epigenetic mechanism—downstream vascular effects remain incompletely defined.	Ascending aneurysm; occasional BAV association	Emerging evidence
*MFAP5*	AD	Likely loss of function	ECM—Impaired microfibril assembly and elastogenesis	Root/ascending aneurysm	Limited evidence
*LTBP3*	AD/AR	Loss of function	Signaling—Altered extracellular TGF-β regulation	Root/ascending aneurysm	Limited evidence

Established and emerging non-syndromic HTAD genes, with associated molecular mechanisms and predominant aortic/vascular phenotypes are summarized in this table. Aortic phenotypes are described according to anatomical distribution. Genes are categorized according to the strength of available evidence (established vs. emerging), reflecting current knowledge and supporting interpretation in clinical and research settings. Abbreviations: AD, autosomal dominant; BAV, bicuspid aortic valve; ECM, extracellular matrix; HTAD, heritable thoracic aortic disease; PDA, patent ductus arteriosus; VSMC, vascular smooth muscle cell.

**Table 3:** Genotype-informed clinical implications and management considerations in pediatric HTAD.

Condition/Gene	Aortic/Vascular Clinical Features and Red Flags	Implications for Management
Marfan syndrome (*FBN1*)	Acute events usually preceded by significant dilation.	Aortic surveillance; β-blocker and/or ARB therapy; consider dual therapy in large, progressive, or high-risk aneurysms. Age-specific thresholds exist for severe early-onset disease.
Loeys–Dietz syndrome (*TGFBR1*, *TGFBR2*)	Aggressive, diffuse vasculopathy with arterial tortuosity; dissection may occur at smaller aortic diameters *. *TGFBR2 Arg528* variants are considered high-risk.	Whole-arterial-tree imaging; close surveillance; β-blocker and/or ARB therapy, with early dual therapy considered in high-risk disease. Lower thresholds than MFS; early surgery in high-risk pediatric disease.
Loeys–Dietz syndrome (*SMAD3*, *TGFB2*, *TGFB3*, *SMAD2*)	Generally intermediate-to-milder LDS spectrum; root/ascending involvement with variable distal or extra-aortic disease.	Aortic and extra-aortic arterial surveillance; β-blocker and/or ARB therapy may be considered; management individualized by genotype and growth. Genotype-informed surgical thresholds; generally less aggressive than *TGFBR1*/*TGFBR2*, with *TGFB3* managed at higher diameters.
Shprintzen–Goldberg syndrome (*SKI*)	Aortic involvement generally milder than LDS.	Aortic surveillance; β-blocker and/or ARB therapy may be considered; No genotype-specific pediatric surgical threshold. Expert individualized management.
Vascular Ehlers–Danlos syndrome (*COL3A1*)	Arterial dissection or rupture may occur without marked dilation *; diffuse medium-vessel and intracranial involvement; high iatrogenic vascular risk.	Non-invasive whole-arterial-tree surveillance; avoid unnecessary invasive procedures; medical therapy and prophylactic surgery individualized in an expert center.
Arterial tortuosity syndrome (*SLC2A10*)	Diffuse arteriopathy with marked tortuosity, aneurysms, and stenoses; pediatric onset, dissection uncommon.	Whole-arterial-tree imaging; follow aneurysms and stenoses; medical therapy individualized, with caution if arterial stenoses are present. Pediatric surgery rarely required.
Cutis laxa type 1B (*EFEMP2*)	Severe early-onset vasculopathy, arterial tortuosity, aneurysms, and stenoses.	Early expert-center follow-up; whole-arterial-tree imaging; individualized therapy. No established surgical threshold; consider high-risk LDS surgical criteria.
Meester–Loeys syndrome (*BGN*)	Aortic dilation/dissection; cerebral vascular involvement reported.	Whole-arterial-tree imaging; close pediatric follow-up; no genotype-specific pediatric surgical threshold; expert individualized management.
Smooth muscle dysfunction syndrome (*ACTA2* p.Arg179)	Severe and diffuse early-onset vasculopathy with childhood aortic events at low diameters *; coronary, cerebrovascular, and pulmonary vascular involvement.	Whole-arterial-tree, cerebrovascular, and coronary assessment; β-blocker preferred with low-dose start and close BP/symptom monitoring. High-risk genotype; lower/early-onset surgical thresholds may be considered
*ACTA2* (*other variants*)	Aortic acute events can occur with modest dilation *; possible coronary and cerebrovascular involvement.	Aortic surveillance; consider cerebrovascular and coronary evaluation; β-blocker and/or ARB may be considered. Lower surgical threshold if high-risk features.
*MYH11*	Ascending aortic aneurysm/dissection; patent ductus arteriosus association.	Aortic surveillance; assess for patent ductus arteriosus and family history; β-blocker and/or ARB may be considered. Consider prophylactic surgery at lower diameters than general non-syndromic aortopathy.
*MYLK*	Root/ascending aneurysm with possible distal extension; dissection may occur with little or no prior dilation *.	Surveillance should not rely on diameter alone; individualized follow-up and expert surgical discussion.
*PRKG1*	High dissection risk, including at small aortic diameters *.	Intensive surveillance from adolescence; β-blocker preferred; individualized expert-center management. Prophylactic surgery indicated at age ≥ 17 years if any aortic dilation is present.
*LOX*	Ascending aortic aneurysm/dissection.	Aortic surveillance; β-blocker and/or ARB may be considered; limited pediatric data. Standard non-syndromic surgical threshold; consider earlier intervention with high-risk features.
*FLNA*	Possible systemic vascular involvement and cardiac valve anomalies.	Cardiovascular and neurological evaluation; vascular imaging individualized. Standard non-syndromic surgical threshold; consider earlier intervention with high-risk features.
*BAV*	Common pediatric aortopathy; red flags include rapid aortic growth and significant valve dysfunction.	Monitor valve dysfunction, and aortic growth; β-blocker and/or ARB may be considered if significant dilation or progression. BAV-specific surgical thresholds; concomitant aortic replacement may be considered during valve surgery.

This table summarizes management-changing genotype–phenotype correlations relevant to surveillance, vascular imaging, medical therapy, and prophylactic surgery. It is not intended to reproduce detailed medical therapy and surgical algorithms; operative decisions should follow statements from scientific societies and be individualized in expert aortopathy centers. * Indicates conditions/genotypes in which acute arterial events have been reported at small or only moderately enlarged arterial diameters, or in which diameter alone may underestimate risk. The term “high-risk” is used according to the clinical context of the 2024 AHA scientific statement on pediatric aortopathies [3], referring to familial, genetic, imaging, or clinical modifiers that may justify intensified surveillance or earlier intervention. Abbreviations: AHA, American Heart Association; ARB, angiotensin-receptor blocker; BAV, bicuspid aortic valve; BP, blood pressure; HTAD, heritable thoracic aortic disease; LDS, Loeys–Dietz syndrome; MFS, Marfan syndrome.

## Data Availability

Not applicable.

## Abbreviations

The following abbreviations are used in this manuscript:

ACMG	American College of Medical Genetics and Genomics
AHA	American Heart Association
ARB(s)	Angiotensin Receptor Blocker(s)
BAV	Bicuspid Aortic Valve
CNV(s)	Copy Number Variant(s)
ECM	Extracellular Matrix
ES	Exome sequencing
HTAD	Heritable Thoracic Aortic Disease
LDS	Loeys–Dietz Syndrome
MFS	Marfan Syndrome
MMP(s)	Matrix Metalloproteinase(s)
NGS	Next-Generation Sequencing
TGF-β	Transforming Growth Factor-β
VSMC(s)	Vascular Smooth Muscle Cell(s)
VUS	Variant of Uncertain Significance
vEDS	Vascular Ehlers–Danlos Syndrome
